# Prediction of response to neoadjuvant chemotherapy in patients with muscle-invasive urothelial bladder cancer: role of immune-related gene expression

**DOI:** 10.1007/s00262-025-04135-8

**Published:** 2025-07-30

**Authors:** Hadeer Mahmoud, Abeer M. Abd El-Aziz, Osama Ezzat, Hany Ibrahim Kenawy, Ahmed A. Shokeir

**Affiliations:** 1 Department of Microbiology and Immunology, Faculty of Pharmacy, Mansoura University, Mansoura, Egypt; 2 Urology Department, Urology and Nephrology Center, Faculty of Medicine, Mansoura University, Mansoura, Egypt; 3 Urology and Nephrology Center, Mansoura University, Mansoura, Egypt

**Keywords:** Bladder cancer, Neoadjuvant chemotherapy, Gene expression, Immunohistochemistry

## Abstract

This prospective study aimed at investigating the role of immune-related gene expression (GATA3, METTL3, ERCC2, PD-L1 and IFN-γ) in the prediction of response to platinum-based neoadjuvant chemotherapy (NAC) in treatment of muscle-invasive bladder cancer (MIBC). A total of 112 patients received four cycles of systemic cisplatin-based NAC and then were subjected to radiological and histopathological evaluation through taking bladder biopsy before surgery. Quantitative real-time RT-PCR assessed GATA3, METTL3 and ERCC2 mRNA expression in tissue samples. Immunohistochemical (IHC) staining was done for GATA3, PD-L1 and IFN-γ in tissue samples. Out of the 112 patients, only 104 completed the proposed protocol of systemic NAC and were included in the final analysis. Out of 104 patients, 43 (41.4%) were responders and the remaining 61 patients (58.6%) were assigned as non-responders. GATA3 and IFN-γ expressions were significantly higher in tumor tissue of the responders compared to non-responders. METTL3 and ERCC2 expressions were significantly lower in tumor tissue of the responders compared to non-responders, whereas, PD-L1 staining showed no significant difference between both groups. Our study suggests that GATA3, METTL3, ERCC2, and IFN-γ could serve as predictive biomarkers for NAC response in MIBC patients. Higher GATA3 and IFN-γ along with lower METTL3 and ERCC2 levels are positively correlated with better response to NAC.

**Supplementary Information:**

The online version contains supplementary material available at 10.1007/s00262-025-04135-8.

## Introduction

Bladder cancer (BC) is ranked ninth in worldwide cancer incidence (7th among men and 17th among women). Approximately 165,000 people die from BC annually [1].

For muscle-invasive bladder cancer (MIBC), radical cystectomy (RC) plus pelvic lymph node dissection remains the most effective treatment. Neoadjuvant chemotherapy (NAC) has been shown to improve cancer-specific survival (CSS) by 5–10% in studies published recently when applied to T_2_*–*T_4_ tumor stages. To identify patients more likely to react to NAC and to shift toward alternative methods in patients more likely to fail, more accurate classification of patients receiving NAC is required [2].

The tumor immune microenvironment has a significant role in anticancer medication [3]. The microenvironment can accurately assess a patient’s prognosis and define the aggressiveness, appearance, function, and response to treatment of tumoral cells [4].

Many studies have been conducted in order to predict the response to NAC using molecular biomarkers that may be determined in the tumor tissue of MIBC patients. Among these are genes including GATA3, METTL-3, PD-L1, IFN-γ, and ERCC2 [5, 6]. MIBCs expressing GATA-binding protein 3 (GATA3) were shown to be less aggressive. The transcription factor GATA3 regulates the expression of genes involved in the luminal differentiation of urothelial epithelial cells [7]. Somatic mutations in methyltransferase-like 3 (METTL3) were associated with disease- or progression-free longer survival compared to wild-type METTL3. These findings suggest that these mutations may be used to forecast how MIBC patients would respond to NAC [8].

Programmed death-ligand 1 (PD-L1) and interferon-γ (IFN-γ) may serve as potential biomarkers for predicting the prognosis and therapeutic response in MIBC patients receiving NAC [5, 9]. Also, somatic deleterious mutations in excision repair cross complementation group 2 (ERCC2) are correlated with pathological response to NAC in MIBC [10].

These investigations were all retrospective or only involved a small number of patients. So, in order to save some patients from needless NAC toxicity and to develop a customized approach for NAC in MIBC patients, it is now necessary to investigate the prior findings in a prospective study involving a larger number of patients in order to predict response to NAC based on genetic and molecular analysis of the tumor tissue. The present study was designed to achieve the above-mentioned target.

## Patients and methods

This is a non-concurrent cohort study carried out at Urology and Nephrology Center, Mansoura University, Mansoura, Egypt, between November 2022 and April 2024 including 112 patients with MIBC, who were histopathologically diagnosed as urothelial tumor by taking bladder biopsy (1st biopsy) through transurethral resection of bladder tumor (TURBT). In all included cases, the TURBT procedure was performed as a biopsy-only procedure for just histopathological correlation without maximum resection of the tumor. We did not include patients who underwent maximum or complete resection in our study to avoid any subsequent selection bias.

All patients received NAC with no concurrent immunotherapy regimens and then were subjected to radiological (MRI) and histopathological evaluation through taking bladder biopsy (2nd biopsy) before RC surgery. Adjacent normal tissue samples were collected from the same patients and were used as control for the tumor samples.

### Inclusion criteria

Patients histologically proven as muscle-invasive urothelial carcinoma of the urinary bladder, should have T_2–4_, N_0–2_ and M_0_ disease and eligible for systemic cisplatin-based NAC with post-chemotherapy check by MRI and histopathologically through cystoscopic biopsy to assess patients’ response before any further treatment.

### Exclusion criteria

Evidence of distant metastasis, prior chemotherapy for same disease, patients who did not complete four cycles of NAC and those who have contraindication for cisplatin-based regimen.

### Evaluation outcome

Patients were classified into two groups according to their response to NAC as follows:

1. Group A (responders) classified as: complete response (those who showed complete disappearance of any evidence of disease radiologically (MRI) and histopathologically) and partial response (those who showed downstaging namely regression of tumor).
2. Group B (non-responders) classified as: static (those who still have MIBC) and progressive (those who showed progressive course of the disease or developed systemic metastasis while receiving treatment).

## Methods

All demographic patients’ criteria were recorded in addition to medical and surgical history, clinical examination, laboratory investigations, and radiological tumor staging.

### Protocol of NAC treatment

The broad line of treatment policy in this study was to give all the eligible patients four cycles of systemic cisplatin-based NAC then restaging of the disease using the same radiological (MRI) and histopathological staging tool to evaluate the degree of response.

Every cycle included:

- Gemcitabine 1000 mg/m^2^ IV over 30 min on days 1 and 8.
- Cisplatin 70 mg/m^2^ IV over 60 min on day 1 following gemcitabine.
- The cycle was repeated every 3 weeks [11].

### Definitive treatment

NAC is typically administered to patients with MIBC who are initially planned for definitive treatment (either RC or radiotherapy). Patients who were non-responders and partial responders to NAC underwent RC as a definitive treatment. Meanwhile, those who achieved complete response preferred the noninvasive radiotherapy instead of surgical treatment to avoid the surgical morbidity and preserve their quality of life. Consequently, they did not undergo RC after shared decision-making with the physician. Yet, they were included in our intention to treat analysis as part of the sample size calculated based on our primary outcome measure (response to NAC, not subsequent definitive therapy).

### Molecular studies

Total RNA was extracted from tissue (1st biopsy) using TRIZOL® reagent (catalog no. 15596026) and then converted to cDNA (Viva cDNA synthesis kit, catalog no. cDSK01-100). Quantitative real-time RT-PCR mRNA expression levels of tissue genes (GATA3, METTL3, ERCC2) were performed using Maxima SYBR green master mix (Thermo Scientific, catalog no. K0251). Glyceraldhyde-3 phosphate dehydrogenase (GAPDH) was used as an endogenous RNA reference gene, and the ΔCt technique was used to normalize gene expression to GAPDH expression [12]. The primers used and their corresponding annealing temperatures are listed in Table S1. Forty cycles were performed with thermal cycling conditions included initial denaturation at 95 °C for 15 min, cycle denaturation at 95 °C for 15 s, annealing for 30 s, cycle extension at 72 °C for 30 s and final extension at 72 °C for 5 min.

### Pathological examination

Formalin fixed paraffin-embedded (FFPE) procedures are the most widely used protocol for histopathology globally [13]. Hematoxylin and eosin (H&E) staining was applied to all tissue paraffin sections obtained from BC samples in order to verify the diagnosis [14].

### Immunohistochemistry (IHC)

IHC staining was done for GATA3, PD-L1 and IFN-γ (biopsy 1). Avidin–Biotin complex (ABC) method was used to perform immunostaining using Dako LSAB kit (catalog no. K0675) according to the following steps;

Paraffin Sects. (4 μm) were deparaffinized with xylene and then rehydrated through distilled water. Sections were immersed in 0.5% v/v hydrogen peroxide/methanol for 10 min to block endogenous peroxidase activity. The heat-induced epitope retrieval (HIER) method was used to retrieve the antigen.

After cooling, the tissue sections were washed, covered with blocking solution for 20 min and then incubated overnight at 4 °C with primary antibodies (GATA3 polyclonal antibody diluted 1:100 (Chongqing Biospes Co., catalog no. YPA1589), PD-L1 monoclonal antibody diluted 1:100 (Quartett, catalog no. QR001) and IFN-γ polyclonal antibody diluted 1:200 (ABclonal, catalog no. A12450)).

Sections were incubated with biotinylated secondary antibody for 20 min at 25 °C, washed, incubated with ABC complex/ horseradish peroxidase (HRP) for 20 min at 25 °C and then washed in PBS buffer for 3–5 min.

Sections were incubated with diaminobenzidine tetrahydrochloride (DAB) in dark for 20 min. Slides were rinsed in gently running tap water. Sections were counterstained with hematoxylin, dehydrated using ascending grades of alcohol and xylene and finally mounted with mounting medium.

Scoring of immunohistochemical markers; GATA3 nuclear positivity was assessed with a semiquantitative immuno-score, as defined by Remmele and Stegner [15]. PD-L1 expression of tumor cells was evaluated by modified H score (MHS) [16], and for IFN-γ, the H score (histochemical score) was used [17].

### Statistical analysis and data interpretation

Data analysis was performed by SPSS software, version 26 (SPSS Inc., PASW statistics for windows version 26. Chicago: SPSS Inc.). Qualitative data were described using number and percent. Quantitative data were described using mean ± standard deviation for normally distributed data and median and range for non-normally distributed data after testing normality using Kolmogorov–Smirnov test. Significance of the obtained results was judged at a *P* value < 0.05.

Chi-Square test and Monte Carlo test were used to compare qualitative data between groups as appropriate. Student’s t test was used to compare between two studied groups for normally distributed data. Mann–Whitney U test was used to compare between two studied groups for non-normally distributed data. Receiver operating characteristics (ROC) curve was used to calculate validity (sensitivity and specificity) of continuous variables with calculation of the best cut-off point. Predictive values and accuracy are assessed using cross-tabulation.

Multivariable binary logistic regression analysis was conducted to identify independent predictors of NAC. Variables that showed statistical significance in univariate analysis were entered into the multivariable model. The results were presented as odds ratios (OR) with 95% confidence intervals (CI), and a *P* value < 0.05 was considered statistically significant. The predictive performance of the model was further evaluated using a ROC curve and the corresponding area under the curve (AUC) with 95% CI.

## Results

**Fig. 1:**
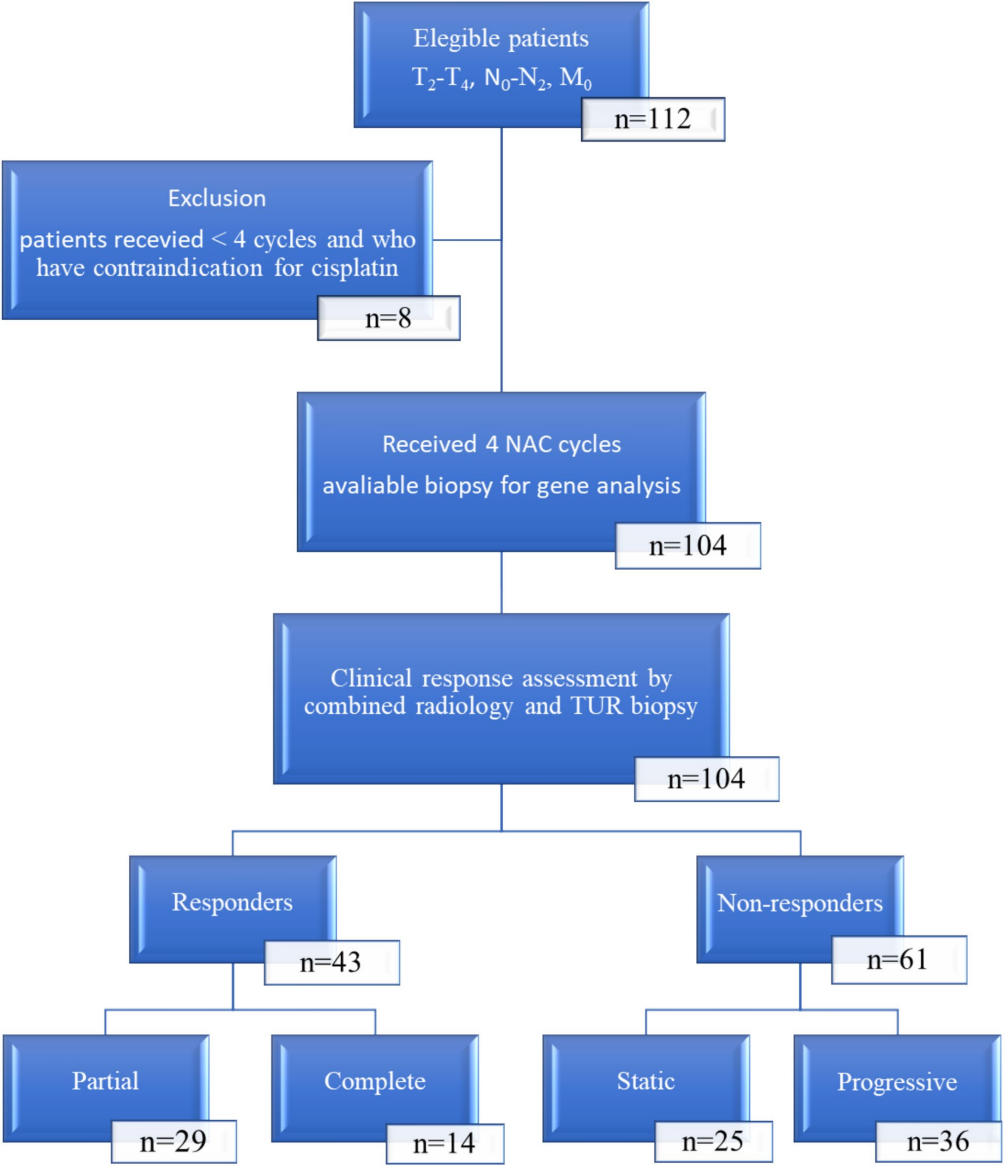
Flowchart of the studied cases

Out of the 112 patients, only 104 had completed the proposed protocol of systemic NAC and included in the final analysis. Out of 104 patients, 43 (41.4%) were responders whether partially (29 patients) or completely (14 patients). The remaining 61 patients (58.6%) were assigned as non-responders whether static (25 patients) or progressive (36 patients). A flowchart of the studied cases is shown in Fig. 1.

Baseline demographic characteristics including age, gender, BMI, smoking status and tumor characteristics were comparable among responders and non-responders as shown in Table 1.

**Table 1:** Demographic characteristics of the studied cases

	Responders (N = 43)	Non-responders (N = 61)	*P* value
Age (years), Mean ± SD	60.67 ± 7.9	62.65 ± 6.2	0.156
*Sex, no. pts. (%)*			
Female	6 (14)	5 (8.2)	0.347
Male	37 (86)	56 (91.8)	
BMI (kg/m^2^), Mean ± SD	28.1 ± 4.1	28.27 ± 4.85	0.852
*Smoking, no. pts. (%)*			
Non-smoker	12 (28)	23 (37.7)	0.290
Smoker	31 (72)	38 (62.3)	
*Tumor stage, no. pts. (%)*			
T2	13 (30.2)	6 (9.8)	0.006
T3	17 (39.5)	20 (32.8)	
T4	13 (30.2)	35 (57.4)	
*N Stage, no. pts. (%)*			
N0	31 (72.1)	34 (55.7)	0.174
N1	7 (16.3)	9 (14.8)	
N2	5 (11.6)	18 (29.5)	
*TUR Pathology, no. pts. (%)*			
Pure	35 (81.4)	47 (77)	0.635
Mixed	8 (18.6)	14 (23)	

SD: standard deviation, BMI: body mass index, no.pts.: number of patients

### Results of gene expression

The results of gene expression analysis of GATA3, METTL3 and ERCC2 genes at baseline by RT-PCR are interpreted as follows:

GATA3 expression was significantly lower in the tumor tissue compared to the control for both responders and non-responders, while the gene expression was significantly higher in tumor tissue of the responders compared to non-responders.

The expression of both METTL3 and ERCC2 was significantly higher in the tumor tissue compared to the control, while the expression of both markers was significantly lower in tumor tissue of responders compared to non-responders as shown in Table 2.

**Table 2:** Comparison of gene expression between tumor and normal tissue in responders and non-responders using quantitative real-time RT-PCR

	Responders (N = 43)		Non-responders (N = 61)				
	Tumor tissue	Control	Tumor tissue	Control	P1*	P2*	P3*
GATA3 RQ, Mean ± SD	0.677 ± 0.100	1.00 ± 0.03	0.320 ± 0.10	1.01 ± 0.04	0.001	0.001	0.001
METTL3 RQ, Mean ± SD	3.03 ± 0.87	1.01 ± 0.04	5.07 ± 1.34	1.0 ± 0.03	0.001	0.001	0.001
ERCC2 RQ, Mean ± SD	2.86 ± 0.68	1.01 ± 0.06	4.88 ± 1.37	0.997 ± 0.07	0.001	0.001	0.001

RQ: Relative Quantitation, P1: responders’ tumor tissue versus control, P2: non-responders’ tumor tissue versus control, P3: tumor tissue in responders versus non-responders, ^*^Student’s t test

**Fig. 2:**
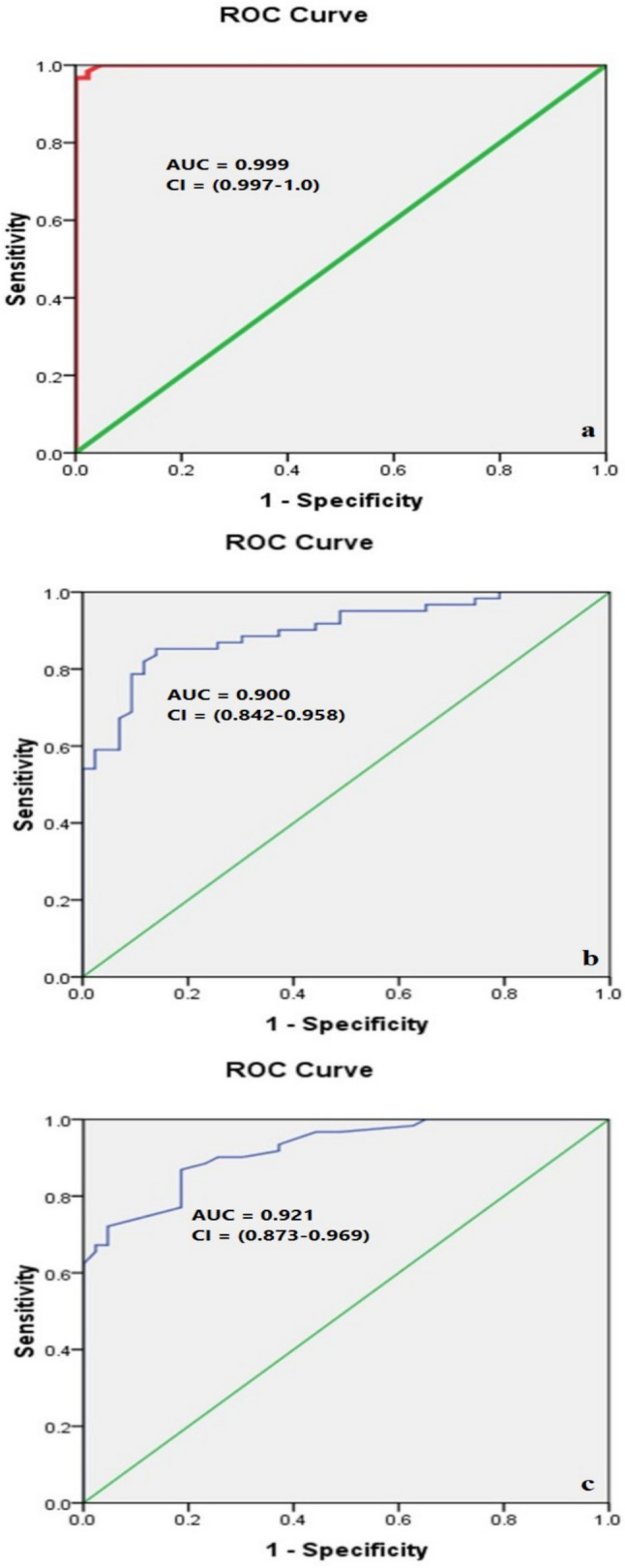
ROC curves for GATA3 (**a**), METTL3 (**b**), and ERCC2 (**c**) genes associated with the response to NAC in MIBC

Table S2 shows the cut-off points that give the best validation of GATA3, METTL3 and ERCC2 in the prediction of the response to NAC based upon ROC curve (Fig. 2).

### Results of immunohistochemical analysis

**Fig. 3:**
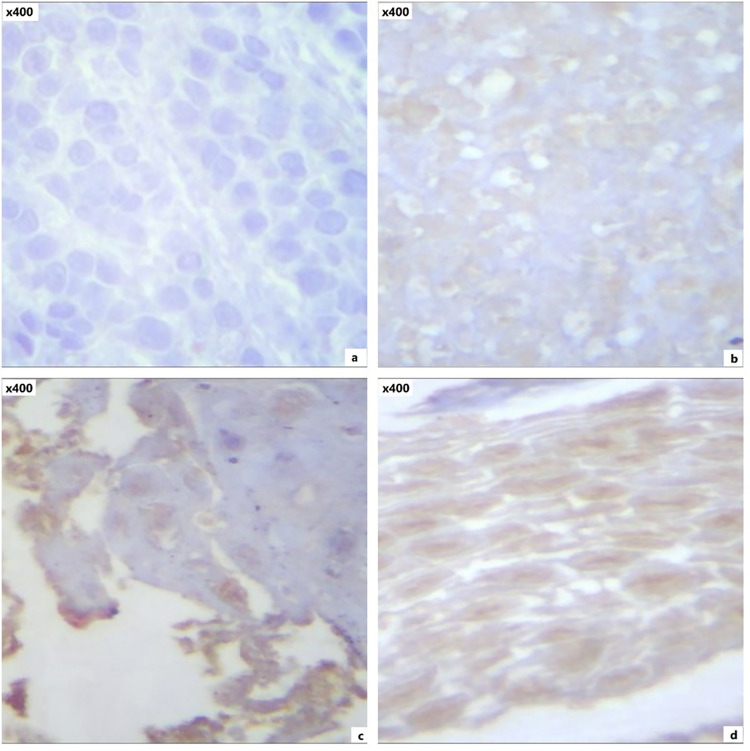
GATA3 staining expression by immunostaining: negative (**a**), weak (**b**), moderate (**c**), and strong (**d**) expression

**Fig. 4:**
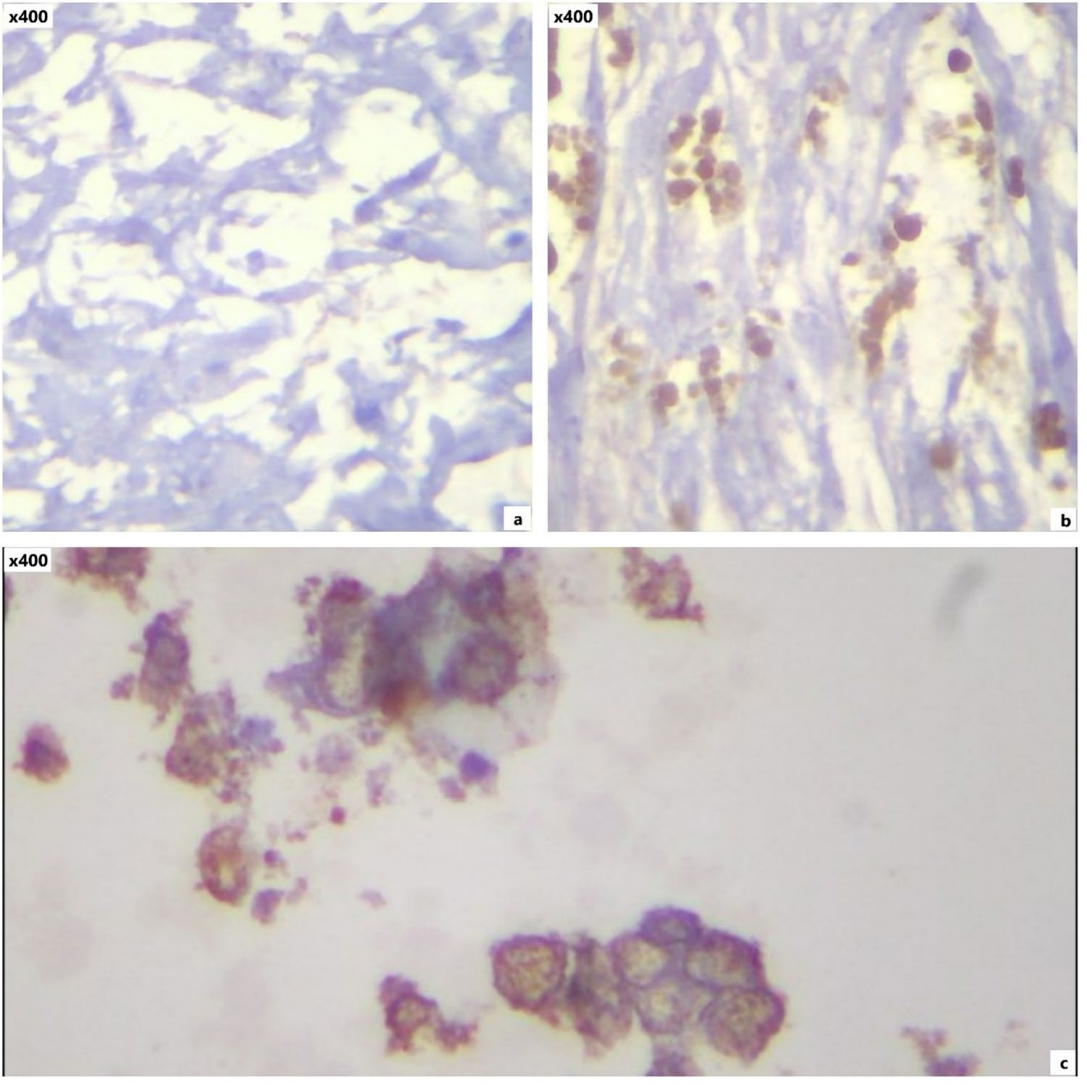
IFN-γ staining expression by immunostaining; negative (**a**), moderate (**b**), and strong (**c**) expression

**Fig. 5:**
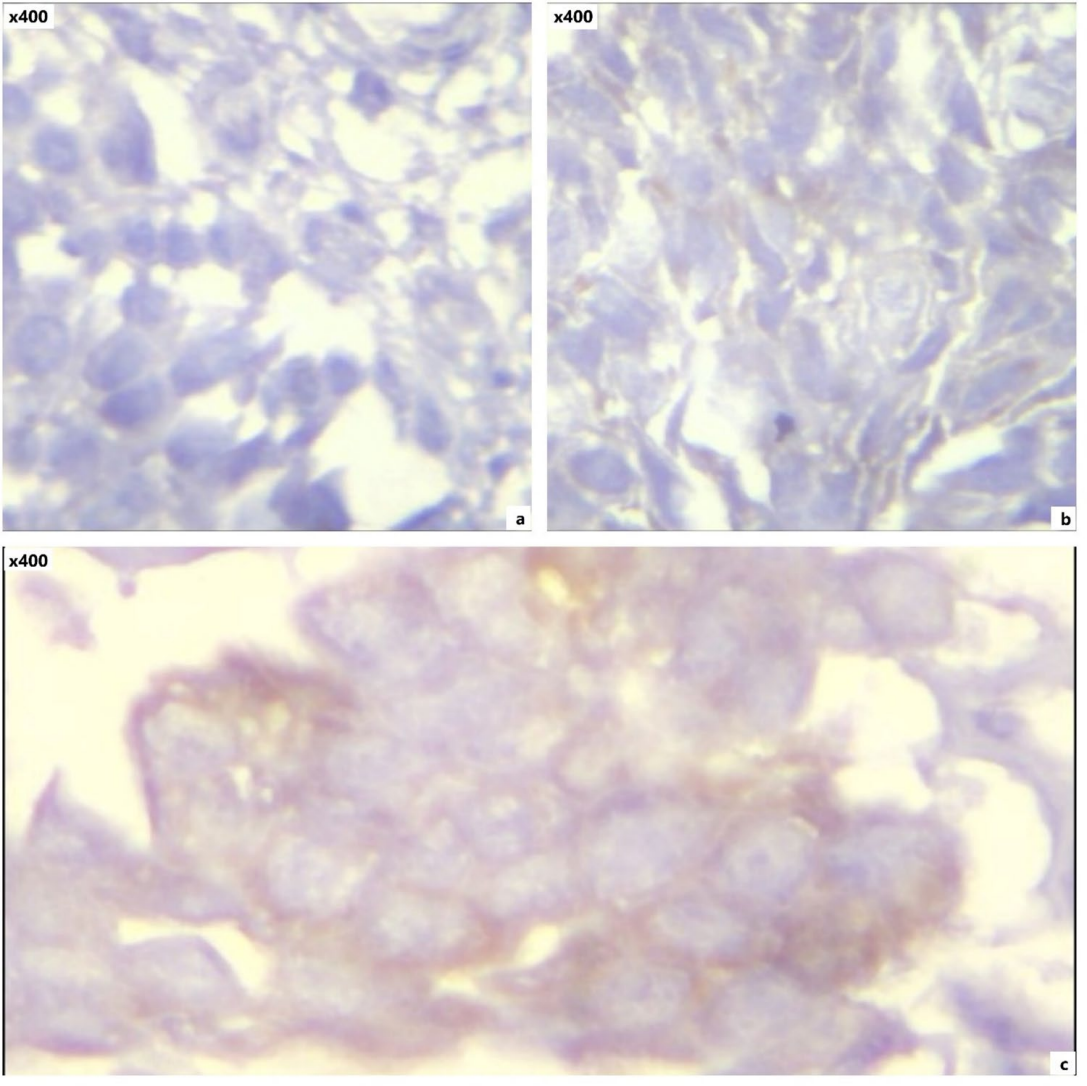
PD-L1 staining expression by immunostaining; negative (**a**), low (**b**), and high (**c**) expression

The immunostaining results revealed that GATA3 (Fig. 3) and IFN-γ (Fig. 4) had significantly higher expression levels in responders than non-responders, while PD-L1 expression (Fig. 5) showed no significant difference between both groups (Table 3).

**Table 3:** Comparison of GATA3, PD-L1, and IFN-γ protein expression in tumor tissue between responders and non-responders using IHC

	Responders (N = 43)	Non-responders (N = 61)	Test of significance
GATA3 staining, Mean ± SD	7.37 ± 2.17	2.72 ± 1.17	t = 14.09 *P* = 0.001
*Strength of expression, no. pts (%)*			
Negative	0	8 (13.1)	*χ*^2MC^ = 53.89
Weak	2 (4.7)	36 (59.0)	*P* < 0.001
Moderate	27 (62.8)	17 (27.9)	
Strong	14 (32.6)	0	
PD-L1 staining, Mean ± SD	109.53 ± 53.04	104.26 ± 47.23	t = 0.53 *P* = 0.297
*Strength of expression, no. pts (%)*			
Negative—low	20 (46.5)	27 (44.3)	*χ*^2^ = 0.051
High	23 (53.5)	34 (55.7)	*P* = 0.82
IFN-γ staining, Mean ± SD	190.23 ± 60.01	53.19 ± 28.18	t = 15.58 *P* = 0.001
*Strength of expression, no. pts (%)*			
Negative	1 (2.3)	22 (36.1)	*χ*^2MC^ = 75.20
Weak	2 (4.7)	34 (55.7)	*P* < 0.001
Moderate	24 (55.8)	5 (8.2)	
Strong	16 (37.2)	0	

*t*: Student t test, *χ*2MC: Monte Carlo test, *χ*2: Chi-Square test

To validate the reliability of the immunohistochemical assessments, inter-reader consistency analysis was performed on 50 samples using Cohen’s Kappa test (Table S3).

**Fig. 6:**
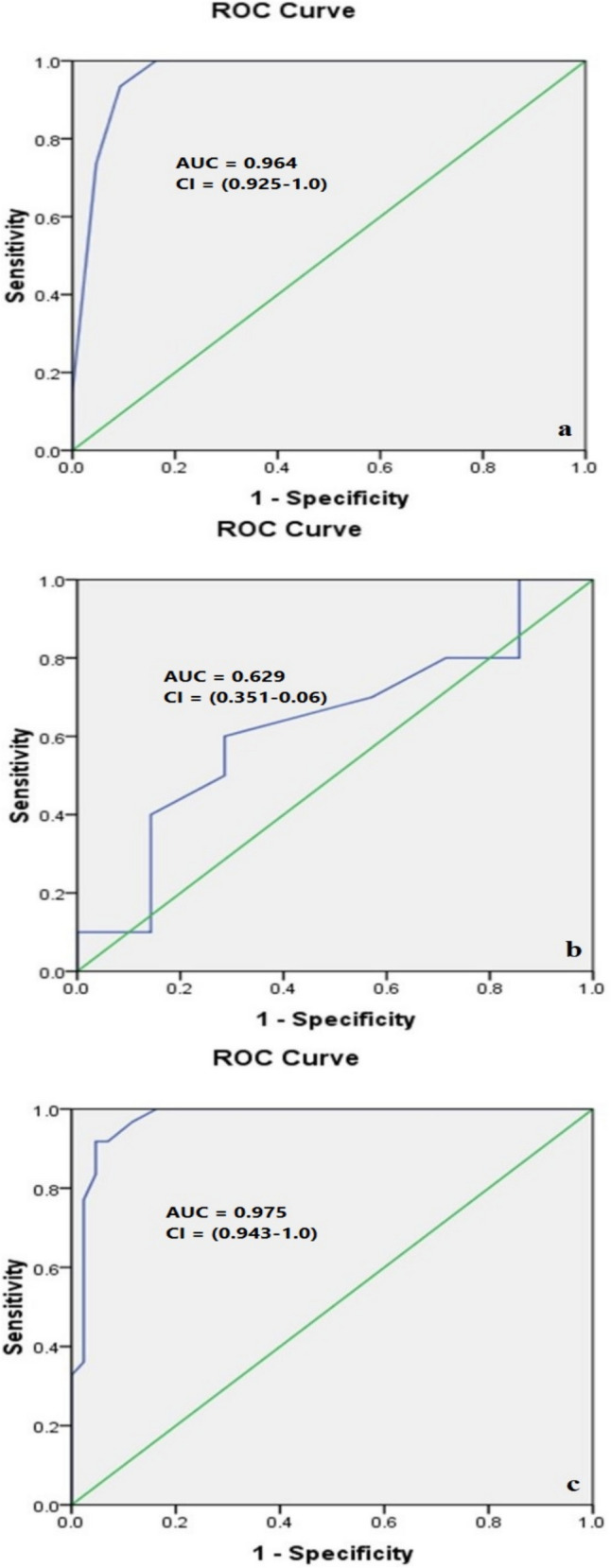
ROC curves of GATA3 (**a**), PD-L1 (**b**), and IFN-γ (**c**) in the prediction of the response to NAC

ROC curve analysis demonstrated that both GATA3 and IFN-γ expression were highly effective in predicting NAC response, with excellent AUC, sensitivity, specificity, positive predictive value (PPV), negative predictive value (NPV), and overall accuracy. Conversely, the PD-L1 expression demonstrated moderate predictive validity (Table S4, Fig. 6).

### Multivariable binary logistic regression analysis

To identify independent predictors of NAC response among the studied biomarkers, a multivariable binary logistic regression analysis was performed. Out of the five biomarkers, four showed statistical significance: ERCC2, METTL3, GATA3, and IFN-γ (*P* < 0.001 for all), whereas PD-L1 showed no significant association (Table S5). The overall prediction accuracy of the model was 98.1%, indicating excellent predictive performance.

**Fig. 7:**
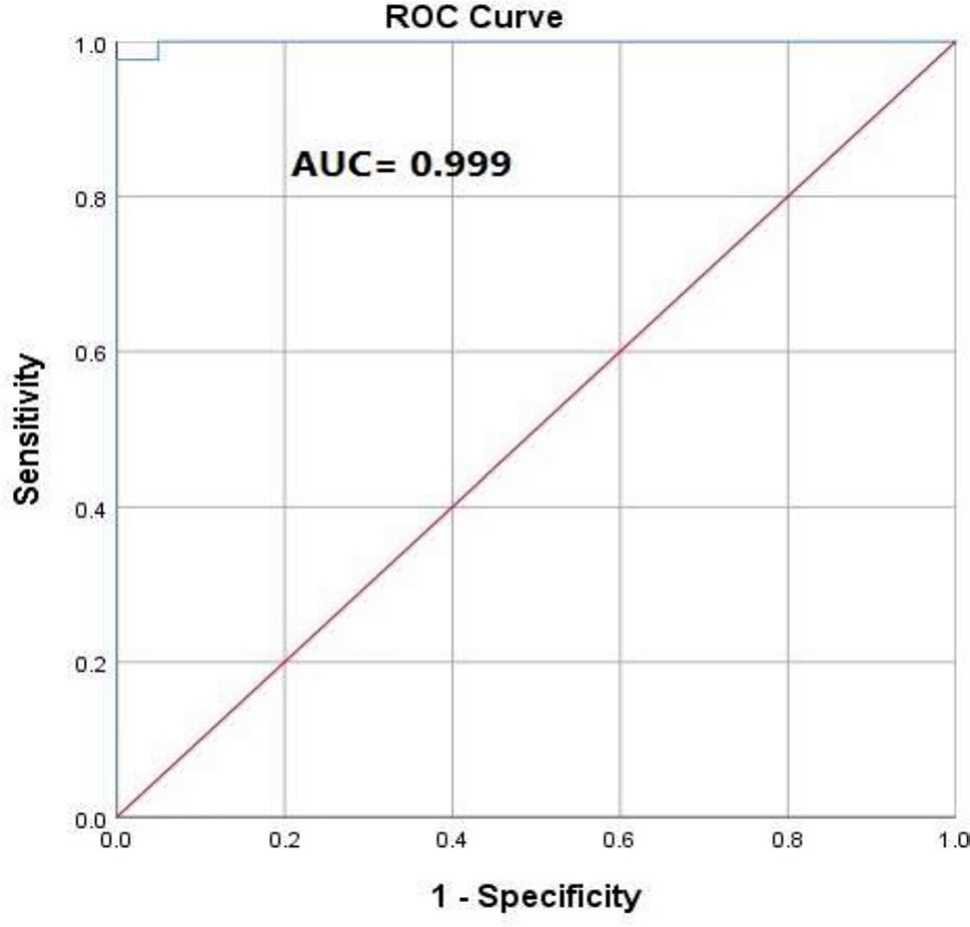
ROC curve for the multivariable logistic regression model combining GATA3, ERCC2, METTL3, and IFN-γ

Furthermore, a ROC curve was generated for the multivariable logistic regression model (Fig. 7), which showed excellent discrimination between responders and non-responders. The curve closely approached the upper left corner, reflecting high sensitivity and specificity. The AUC was 0.99, reflecting the strong predictive performance of the combined biomarker model (Table S6).

## Discussion

Cisplatin-based NAC followed by RC is recommended for patients with MIBC [10]. NAC seeks to lower surgical difficulty, increase patients’ postoperative long-term survival rate, and manage local lesion and distant small tumor metastases. However, in patients who are not responsive to NAC, the ideal operating time will be postponed [18]. The patients who will benefit from NAC cannot be determined by clinicians. Thus, it has been reported that genetic biomarkers correlate with NAC responsiveness [10].

In our study, 41.4% of participants were responders, while 58.6% were non-responders. These results agree with other studies which indicated that the use of NAC improves pathological downstaging and increases patient survival rates in approximately 40% of patients [19].

The transcription factor GATA3 regulates the expression of genes associated with the luminal differentiation of urothelial epithelial cells. Loss of GATA3 expression has been linked to increased tumor cell motility and invasion, emphasizing its role in tumor suppression [7]. This study revealed a significant difference in GATA3 gene expression between tumor cells (both responders and non-responders) and normal control cells. Specifically, we observed markedly lower expression of GATA3 in tumor tissues compared to the control group. This finding aligns with other studies [20] which reinforce the importance of GATA3 as a biomarker for MIBC.

A previous preclinical research demonstrated that GATA3 knockdown causes oncogenic genes to be up-regulated and molecules that are protective against bladder tumorigenesis to be down-regulated, thus suggesting a protective role of GATA3 in BC [20]. In this study, we found that GATA3 expression was significantly higher in responders compared to non-responders, indicating that elevated GATA3 expression is strongly associated with a positive response to NAC. Further analysis using GATA3 staining supported these findings.

Our findings are in agreement with other investigators, which suggested that GATA3 prevents BC progression by inhibiting cell migration and invasion. Thus, GATA3-negative patients have a poorer prognosis, while tumors expressing GATA3 were less aggressive and the patients had higher survival rates [7, 21].

However, our results contrast with some researchers, who suggested that lower GATA3 expression was associated with a better pathological response to NAC [22]. This discrepancy between our findings and those of the others may be attributed to their reliance on bioinformatic analyses on a limited number of patients without in vivo or in vitro experiments.

Previous investigations have demonstrated the crucial role that m6A methylation alterations play in BC. The proliferation and invasion of BC cells was considerably promoted by METTL3 overexpression [23]. METTL3 expression knockdown, on the other hand, significantly decreased BC cell viability, invasion, proliferation, and carcinogenic potential [24].

Our results demonstrated significantly higher METTL3 gene expression in tumor tissues, both in responders and non-responders, compared to normal control cells. These findings are consistent with similar studies, which indicated that METTL3 was upregulated in BC cell lines compared to normal urinary epithelial cells [25].

Our research identifies METTL3 as another predictive biomarker for chemotherapy response. The data show that responders had significantly lower METTL3 expression compared to non-responders indicating that lower METTL3 expression is associated with a better response to chemotherapy. Conversely, higher METTL3 expression may be linked to NAC resistance.

These findings are consistent with a group of authors’ findings, who demonstrated that MIBC patients who had mutant METTL3 exhibited a pathological response to NAC and had significantly longer overall survival or disease/progression-free survival compared to patients who had wild-type METTL3 [8]. Some investigators showed that high METTL3 expression was associated with poor prognosis in BC patients [25].

Cisplatin causes DNA damage, interferes with cell viability and triggers apoptosis. Many studies have been done to investigate the relationship between response to NAC and the mutation of DNA damage repair (DDR) genes, such as ERCC2 [26]. Mutations in ERCC2 cause it to lose its normal function, which makes tumor cells more vulnerable to substances that damage DNA, including cisplatin [27].

Our study demonstrated a significant increase in ERCC2 gene expression in tumor tissues, both in responders and non-responders, compared to normal control cells. This higher expression of ERCC2 in tumor cells is consistent with the findings of other investigators, who reported upregulation of ERCC2 in tumor tissue samples compared to noncancerous tissues [28].

Our study identifies ERCC2 as a predictive biomarker for chemotherapy response in BC patients. The data show that non-responders had significantly higher ERCC2 expression compared to responders and lower ERCC2 expression is associated with a better response to NAC, indicating that ERCC2 plays a crucial role in modulating chemosensitivity. These results agree with similar studies which found higher mutation rates in ERCC2 among responders compared to non-responders to cisplatin-based NAC [29, 30].

Other reports demonstrated that mutations in ERCC2 gene in responders and non-responders did not show an association with pathologic response. This contrast with our results may be due to the use of different platinum-containing chemotherapy regimens [31].

Immunogenic tumors like BC have the ability to interact with immune cell surface checkpoints like PD-1 and PD-L1 and inhibit the immune system. Some studies showed poor outcomes in BC with high levels of PD-L1 expression [32]. Nevertheless, in our study, quantitative analysis revealed no significant difference in PD-L1 expression between responders and non-responders. Additionally, the qualitative analysis showed that the distribution of PD-L1 expression levels did not significantly differ between the groups. These findings suggest that PD-L1 expression may not be a reliable marker for distinguishing between responders and non-responders to NAC in MIBC. Our results are consistent with the findings of other investigators [9, 33] who also reported that PD-L1 does not appear to be a predictive biomarker of response to NAC in BC.

Activation and proliferation of lymphocytes, adhesion and migration of leukocytes, processing and presentation of antigens and cellular response to the IFN-γ are among the most complex biological processes. High inflammatory tumors have higher levels of certain chemokines, which are essential in regulating the immune system’s response to cancer. The complex immune response in MIBC patients involved the upregulation of several IFN-γ-inducible chemokines, MHC class II molecules and the immune checkpoint genes. T-helper 1 (Th1) cells release IFN-γ, which induces the overexpression of these chemokines. CXCR3 chemokine receptor is activated by IFN-γ-induced chemokines, such as CXCL9, CXCL10 and CXCL11. A variant transcript of CXCR3 is associated with the response to NAC and improved overall survival in MIBC [34].

Our study aimed to assess the role of IFN-γ as a predictive biomarker for the response to NAC in BC patients. The quantitative analysis revealed that responders exhibited significantly higher expression levels of IFN-γ compared to non-responders. This significant difference suggests that elevated IFN-γ expression is strongly associated with a positive response to chemotherapy. Conversely, the lower expression levels in non-responders suggest a less effective immune response, which may be associated with chemotherapy resistance. Our findings align with other investigators which reported that IFN-γ expression is highly associated with response to NAC in BC patients [5].

Our study is the first prospective analysis with a large homogeneous sample size to investigate the impact of five key biomarkers (GATA3, METTL3, ERCC2, PD-L1, and IFN-γ) on the response to NAC in patients with MIBC. The identification of these biomarkers provides a foundation for personalized treatment strategies, allowing for better prediction of chemotherapy outcomes and potentially guiding the development of targeted therapies. In addition, we developed a predictive model incorporating the most relevant biomarkers (GATA3, METTL3, ERCC2, and IFN-γ) through multivariable analysis. This model demonstrated strong discriminatory power in identifying responders to NAC, supporting the potential utility of a combined biomarker-based approach in clinical decision-making. 

However, our study is not without limitations. We assessed the impact of PD-L1 and IFN-γ expression using IHC alone without gene expression which may not fully capture the complexity of these biomarkers’ roles in predicting NAC response. Moreover, although our study identified significant associations between GATA3 and METTL3 expression levels and NAC response, the exact mechanisms by which these biomarkers influence chemotherapy sensitivity and resistance remain unclear. In addition, our study was conducted at a single center, which may limit the reproducibility of the findings. Multi-center studies involving diverse patient populations are needed to validate our results and ensure they are applicable to broader clinical settings.

## Conclusion

Higher expression of GATA3 and IFN-γ along with lower expression of METTL3 and ERCC2 was positively correlated with better response to NAC and more favorable outcomes. The combination of these four biomarkers formed a novel predictive model for NAC response. In contrast, PD-L1 did not demonstrate predictive utility.

## Supplementary Information

Below is the link to the electronic supplementary material.

Supplementary file1 (DOCX 26 KB)

## Data Availability

The datasets generated or analysed during the current study are available from the corresponding author on request.
