# Supplementary material for: Prediction of response to neoadjuvant chemotherapy in patients with muscle-invasive urothelial bladder cancer: role of immune-related gene expression
Source: Cancer Immunol Immunother. 2025 Jul 30;74(9):279. doi: 10.1007/s00262-025-04135-8 (PMC12311079; doi:10.1007/s00262-025-04135-8)
Supplement: Supplementary file 1 — Supplementary file1 (DOCX 26 KB) [file 262_2025_4135_MOESM1_ESM.docx]

**Table S1. Primers’ sequences and their corresponding annealing temperatures used for quantitative real-time RT-PCR**

| **Primer** |  | **Sequence** | **Annealing temperature** |
| --- | --- | --- | --- |
| GAPDH | Forward | 5′-ACCACAGTCCATGCCATCCAC-3′ | 59 ℃ |
|  | Reverse | 5′-TCCACCACCCTGTTGCTGTA-3′ |  |
| GATA3 | Forward | 5′-TCATTAAGCCCAAGCGAAGG-3′ | 57 ℃ |
|  | Reverse | 5′-GTCCCCATTGGCATTCCTC-3′ |  |
| METTL3 | Forward | 5′-CAAGCTGCACTTCAGACGAA-3′ | 56 ℃ |
|  | Reverse | 5′-GCTTGGCGTGTGGTCTTT-3′ |  |
| ERCC2 | Forward | 5′-GGGTCATGGAGTCCTGGAGA-3′ | 59 ℃ |
|  | Reverse | 5′-CCATCGACGTCCTTCCCAAA-3′ |  |

**Table S2. Validation of GATA3, METTL3 and ERCC2 gene expression in the prediction of the response to NAC**

|  | **AUC (95%CI)** | **P value** | **Cut off point** | **Sensitivity %** | **Specificity%** | **PPV%** | **NPV%** | **Accuracy%** |
| --- | --- | --- | --- | --- | --- | --- | --- | --- |
| **GATA3** | 0.999 (0.997-1.0) | 0.001 | ≤ 0.515 | 98.4 | 97.7 | 98.4 | 97.7 | 98.1 |
| **METTL3** | 0.900 (0.842-0.958) | 0.001 | ≥ 3.72 | 85.2 | 86.0 | 89.7 | 80.4 | 85.6 |
| **ERCC2** | 0.921 (0.873-0.969) | 0.001 | ≥ 3.55 | 86.9 | 81.4 | 86.9 | 81.4 | 84.6 |

^AUC^: area under curve, ^PPV^: positive predictive value, ^NPV^: negative predictive value

**Table S3. Reliability of Immunohistochemical Assessments: Cohen’s Kappa Analysis**

| **Biomarker** | **Cohen’s Kappa Score** | **Agreement Level** |
| --- | --- | --- |
| **GATA3** | 0.775 | Substantial Agreement |
| **PD-L1** | 0.826 | Almost Perfect Agreement |
| **IFN-γ** | 0.855 | Almost Perfect Agreement |

**Table S4. Validation of GATA3, PD-L1 and** **IFN-γ protein expression in the prediction of the response to NAC**

|  | **AUC (95%CI)** | **P value** | **Cut off point** | **Sensitivity %** | **Specificity%** | **PPV%** | **NPV%** | **Accuracy%** |
| --- | --- | --- | --- | --- | --- | --- | --- | --- |
| **GATA3** | 0.964 (0.925-1.0) | 0.001 | ≤ 4.5 | 93.4 | 90.7 | 93.4 | 90.7 | 92.3 |
| **PD-L1** | 0.629 (0.351-0.06) | 0.380 | ≥ 95 | 60.0 | 71.4 | 75.0 | 55.6 | 64.7 |
| **IFN-γ** | 0.975 (0.943-1.0) | 0.001 | ≤ 125 | 98.4 | 86.0 | 90.9 | 97.4 | 93.3 |

^AUC^: area under curve, ^PPV^: positive predictive value, ^NPV^: negative predictive value

**Table S5. Binary logistic regression analysis of predictors of response among the studied cases**

|  | β | P value | Odds ratio  (95%CI) |
| --- | --- | --- | --- |
| ERCC2 RQ | -1.59 | 0.001* | 0.202 (0.110-0.372) |
| METTL3 RQ | -2.34 | 0.001* | 0.097 (0.039-0.243) |
| GATA3 antibody | 1.64 | 0.001* | 5.14 (2.59-10.17) |
| PD-L1 antibody | -0.002 | 0.623 | 0.998 (0.989-1.01) |
| IFN-γ antibody | 0.061 | 0.001* | 1.06 (1.04-1.09) |
| Overall correct % predicted = 98.1 | | | |

**Table S6. ROC curve analysis showing the diagnostic accuracy of the combined biomarkers in predicting response to NAC**

|  | AUC  (95%CI) | P value | Sensitivity | Specificity | PPV | NPV | Accuracy |
| --- | --- | --- | --- | --- | --- | --- | --- |
| Combined markers | 0.999  (0.996-1.0) | 0.001* | 97.7% | 95.1% | 93.3% | 98.3% | 96.2% |

Used cut off point from predicted probability
